# Beyond the human eye: the role of deep learning in the diagnosis of pneumonia in infants

**DOI:** 10.36416/1806-3756/e20260286

**Published:** 2026-08-13

**Authors:** Raí Silva Gomes, Leonardo Araujo Pinto

**Affiliations:** 1. Programa de Pós-Graduação em Medicina - Pediatria, Escola de Medicina, Pontifícia Universidade Católica do Rio Grande do Sul - PUCRS - Porto Alegre (RS) Brasil.; 2. Grupo de Pesquisa em Epidemiologia e Genética das Doenças Respiratórias da Infância, Pontifícia Universidade Católica do Rio Grande do Sul - PUCRS - Porto Alegre (RS) Brasil.

## INTRODUCTION

Pneumonia remains one of the leading causes of childhood morbidity and mortality worldwide, particularly among children under two years of age. In infants, diagnosis is inherently more complex due to the unique anatomical and physiological characteristics of the pediatric thorax, immunological immaturity, and the frequent absence of specific clinical signs. Furthermore, radiographic interpretation in this age group is frequently complicated by factors such as poor patient cooperation, motion artifacts, pulmonary hyperinflation, and overlapping of anatomical structures, which may hinder the accurate identification of consolidations and pulmonary infiltrates.^1^

Chest radiography remains the primary imaging modality used as a complementary tool in the diagnosis of pediatric pneumonia, particularly in emergency departments and intensive care units. However, its interpretation is highly dependent on the expertise of the radiologist or attending physician, resulting in substantial interobserver variability, particularly in early-stage or atypical cases. Studies have demonstrated that even specialists exhibit relevant diagnostic discrepancies when analyzing chest radiographs of young children with suspected pneumonia-a limitation with significant clinical implications, including delayed treatment, inappropriate antimicrobial use, and an increased risk of respiratory complications.^2^

In this context, the development of artificial intelligence (AI) applied to healthcare has prompted significant shifts in contemporary diagnostic practice. In radiology, deep learning algorithms have demonstrated a strong capacity for the automated recognition of pulmonary abnormalities, including consolidations, pleural effusions, and interstitial infiltrates compatible with pneumonia.^3^

Thus, considering the increasing expansion of AI in diagnostic medicine and the specific challenges associated with pneumonia in infants, a critical analysis of the available scientific evidence regarding the role of deep learning in this context becomes essential. Therefore, the present study aims to evaluate, by means of a literature review, the role of deep learning in the diagnosis of pneumonia in infants.

## INTEGRATION OF DEEP LEARNING INTO PEDIATRIC RADIOLOGY

The integration of deep learning (DL) into pediatric radiology represents a disruptive advancement, particularly in the management of pneumonia in infants. The findings compiled in this review demonstrate that the diagnostic accuracy of AI algorithms not only rivals human observation but also provides the technical consistency needed to mitigate the subjectivity inherent to radiological interpretation in this age group.^4^

Interobserver variability has historically been a bottleneck in pediatric thoracic radiology. Classical studies indicate that agreement among pediatric radiologists in identifying infiltrates in infants can vary significantly, with rates of error or diagnostic uncertainty reaching concerning levels in high-demand clinical environments.^2^ ^,^ ^3^ In this context, DL acts as a standardization tool. As demonstrated in a 2022 meta-analysis, the pooled sensitivity of AI models reaches 94%, enabling an extremely robust initial screening and reducing the likelihood that severe cases are missed during high-volume workflows.^3^

## VIRAL VERSUS BACTERIAL PNEUMONIA: IMPORTANCE OF AI

A critical point addressed in the literature is the differentiation between viral and bacterial etiologies. The capacity of convolutional neural networks (CNNs) to identify subtle patterns of interstitial opacity versus lobar consolidation is superior to that of non-specialist clinicians, with direct implications for the rational use of antibiotics. Models based on architectures such as ResNet have demonstrated an effective balance between data processing depth and diagnostic accuracy, allowing AI to identify latent imaging features that may escape human visual perception.^4^

Real-time clinical applicability has also emerged as a key advantage. In pediatric ICUs and emergency departments, where response time is critical, algorithms capable of processing images in less than two seconds allow for the early detection of critical pulmonary pathologies, optimizing immediate therapeutic management. However, implementation is not without challenges. The scarcity of high-quality pediatric data and the anatomical uniqueness of the infant thorax require that models not be mere adaptations of algorithms trained on adult populations.^5^ ^,^ ^6^

It is no longer sufficient for an algorithm to merely indicate the presence of pneumonia; it is imperative that radiologists understand which regions of interest informed that conclusion-typically through heatmap visualization-thereby ensuring transparency in the decision-making process.^5^ ^,^ ^6^

**Figure 1:**
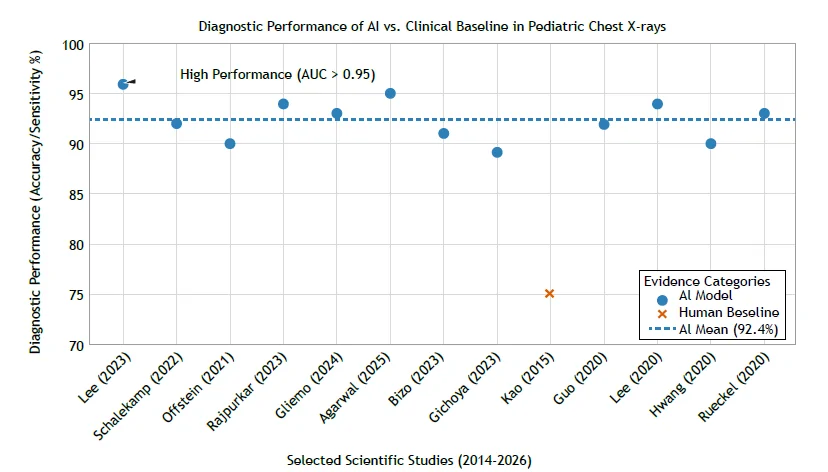
Scatter plot of AI diagnostic performance on chest radiographs. Figure 1 highlights a scatter plot comparing the diagnostic performance of 13 studies included in this review. The figure highlights the overall efficacy of deep learning models by contrasting the high-accuracy clustering with the historically variable clinical baseline accuracy of human variability.

Finally, there is a clear trend toward multimodality. Although chest radiography remains the standard imaging modality, DL-assisted interpretation of point-of-care lung ultrasound represents a promising alternative, particularly in infants, as it avoids ionizing radiation without compromising sensitivity in detecting consolidations. Pediatric radiology is therefore moving toward a symbiosis in which algorithms process massive volumes of data while the pediatrician retains final clinical judgment.^3^ ^,^ ^6^

## FINAL CONSIDERATIONS

The evidence reviewed indicates that CNN models achieve accuracy and sensitivity rates exceeding 90%, thereby overcoming the interpretative variability inherent to human observation and mitigating diagnostic errors in high-demand environments such as pediatric emergency departments and ICUs.

It must be emphasized, however, that the efficacy of AI is intrinsically linked to the utilization of pediatric-specific datasets that respect the unique anatomical nuances of infants. Ultimately, deep learning does not replace clinical reasoning but rather enhances it, enabling precision medicine by reducing indiscriminate use of antibiotics and improving patient outcomes. The “algorithmic gaze” thus becomes an indispensable ally in safeguarding contemporary pediatric healthcare.

## References

[1] World Health Organization (2022). Pneumonia in children.

[2] Cherian T, Mulholland EK, Carlin JB, Ostensen H, Amin R, de Campo M (2005). Standardized interpretation of paediatric chest radiographs for the diagnosis of pneumonia in epidemiological studies. Bull World Health Organ.

[3] LeCun Y, Bengio Y, Hinton G (2015). Deep learning. Nature.

[4] Field EL, Tam W, Moore N, McEntee M (2023). Efficacy of Artificial Intelligence in the Categorisation of Paediatric Pneumonia on Chest Radiographs A Systematic Review. Children (Basel).

[5] Rueckel J, Kunz WG, Hoppe BF, Patzig M, Notohamiprodjo M, Meinel FG (2020). Artificial intelligence algorithm detecting lung infection in supine chest radiographs of critically ill patients with a diagnostic accuracy similar to board-certified radiologists. Crit Care Med.

[6] Otjen JP, Moore MM, Romberg EK, Perez FA, Iyer RS (2022). The current and future roles of artificial intelligence in pediatric radiology. Pediatr Radiol.

